# Obituary

**Published:** 1871-05

**Authors:** 


### Obituary.

***E. A. Clark, M.D.,***

Professor Principles of Surgery and Surgical Anatomy in the Missouri Medical College, died suddenly at the residence of his parents, Paris, Ill., on the 16th ult. Dr. Clark graduated at Rush Medical College in 1861, served with distinction as Surgeon U. S. V. during the late war, and had achieved a position of eminence in the profession, although, we believe, not yet thirty-five years of age at the time of his decease. He was quite generally known by his contributions to the medical press, and the news of his untimely demise will be received by the general regret of the profession as well as by his more immediate friends and acquaintances whose affection and respect he had secured by his many excellent qualities both of heart and head.

***Dr. Felix Von Niemeyer,***

Director of the Medical Clinic of the University of Tübingen, died on the 14th of March. He was, perhaps, the most eminent of the present class of German pathologists, and had recently become familiar to the pro fession of this country by his excellent volumes on the Elements of Practical Medicine. His death is said to have been accelerated by the onerous character of his duties during the recent Franco-Prussian war.

***J. E. Scanland, M.D.,***

Of this city, was killed a few weeks since. Dr. S. graduated at Rush Medical College with distinction, February, 1868. Subsequently, having engaged in private practice he was occupied a portion of the time as one of the instructors in the spring session of the College, in which position he gave excellent satisfaction. During the present session he had not been connected with the institution.

At the time of his death, Dr. S. was about 29 years of age, of commanding physique, courtly manners, and the bearing of a polished gentleman. As such, he was a general favorite in the circle of his acquaintance. Indirectly, these qualities were the cause of his untimely death, which took place at the hands of the brother of Mrs. Scanland. Whilst the case is pending in the courts, it is of course improper for the press to utter any word which may prejudice the prisoner's case, or in anywise interfere with the impartial administration of justice. The facts, about which there seems no dispute, are, that some intermeddling villain had endeavored to poison the mind of Mrs. S. with jealousy of her husband. Whether rightfully or wrongfully, Dr. S. supposed this intermeddler his brother-in-law, and while remonstrating with him against his presumed assumption of the role of a detective, and possibly indulging in violent language, he was, although wholly unarmed and defenceless, shot down (on the plea of self-defence) and instantly killed. The interview and the shooting took place in broad daylight, and upon an unobstructed sidewalk of one of our principal streets. So far as the immediate cause of the difficulty was concerned, there seems no reason to doubt that Dr. S. was wholly free from imputation, and the lady whose name was most outrageously dragged into the case was thereby most vilely wronged.

The recent conviction of Mrs. Fair, in San Francisco, lends strength to the hope that our courts are awaking to a realizing sense of the value of human life, and are disposed to throw some safeguards around it. There is a sentimental brutality abroad which satiates itself in sympathy with those who violently "put out the light" of life, but has no tears or even "regrets" for the victims of murder, "most foul and unnatural."

We trust that in the present case the homicide may be *fairly* investigated.

[Since the above was put in type, the Grand Jury have discharged the prisoner as acting in *self-defence.* The more probable reason is, that in Chicago it is considered no crime to shoot a doctor, or any other man, if there is a woman in the case.]

### *J. Wolcott Hooker,*

Who for a few months attempted the publication of this JOURNAL in 1869, and, as many of our old subscribers know to their cost, failed to issue the same after a brief effort in that direction, and through whose instrumentality several thousands of dollars were lost to the present writer (to his continuously increasing disgust)—having disappeared from our knowledge and anxious view for the last eighteen or twenty months, has recently been heard from as the principal and, indeed, sole, actor in a somewhat startling tragedy. J. W. H. committed suicide at St. Louis, April 18th, by hanging himself with a trunk strap attached to a stout iron staple of the window casing of his room. It seems he made the preparations for his exit with remarkable care, and an evident eye to its tragic effect. We forgive him now, and say, Peace to his ashes.

### *Hon. Harrison Noble, M.D.*

Died, at his residence near Heyworth, Illinois, on the 12th of August, 1870, Hon. HARRISON NOBLE, M.D., in the 59th year of his age.

Dr. Noble graduated at the Ohio College of Medicine in the spring of 1847. Previously to this time he had been engaged in agricultural pursuits, and early in the history of McLean county had been county surveyor. He was a man of great strength of mind and will—a close student—and immediately after his debut in medicine became prominent in the profession of the State; at one time being elected President of the Illinois State Medical Society, and was one of its most honored and efficient presiding officers. He was regular in his attendance upon the meetings of the State Society, acting as chairman of the committee upon the practice of medicine, and other important committees At one time he made a special report upon the subject of typhoid fever, that was regarded as a very able effort. He was also a member of the American Medical Association, and frequently attended its sessions.

The doctor represented his district in the State legislature two consecutive terms, and was a prominent working member of that body. The then Governor of the State declared in a public speech that he owed more to him for valuable assistance than any other member of either house.

At the last gubernatorial canvass he was a prominent candidate for the nomination for Governor, by a number of the central counties of the State.

Personally, Dr. Noble was a man of splendid physique and commanding presence—a good adviser, and a true and faithful friend. By his death, a void has been created in the circle of society he ornamented, and in the profession that he elevated by his counsels; but,

"He gave his honors to the world again,
His blessed part to Heaven, and slept in peace."

COM.

---

At a called meeting of the McLean County Medical Society, held at the office of Drs. Bradley & Laughlin, in Bloomington, Ill., on the 15th day of March, 1871, to take action with regard to the death of Dr. S. W. NOBLE, the following preamble and resolutions were unanimously adopted.

R. D. BRADLEY, *Sec.*

WHEREAS, We learn with sadness that our much loved companion, Dr. S. W. NOBLE, after a protracted and painful illness—which he endured with great fortitude and patience—died on yesterday afternoon, at 7 o'clock:

*Resolved*, That in his untimely death, our Society has lost one of its most honored and useful members, the community a man of superior character and influence, and his family a devoted husband and affectionate father.

That we will ever cherish the memory of his ardent attachment to the interests of our Society, and his unremitting devotion to the cause of our noble profession, to whose dignity and practice he consecrated all his powers, and all his great talents—and in the service of which he spent his life.

That we commend Dr. Noble's example, in that his devotion to his beloved science prompted him to express his desire that a post mortem examination of his body be made, in order to confirm or correct the diagnosis of his case.

That we hereby tender to his stricken and bereaved family and friends, our sincere sympathy in this their sad and irreparable loss; and that a copy of these resolutions be published in the city papers, in the CHICAGO MEDICAL JOURNAL, and in the *Western Lancet and Observer*, and that a copy be handed to the afflicted wife of our deceased brother.

A. H. LUCE,
R. G. LAUGHLIN,
T. F. WORRELL,
*Committee.*

